# Whole exome sequencing identified mutations causing hearing loss in five consanguineous Pakistani families

**DOI:** 10.1186/s12881-020-01087-x

**Published:** 2020-07-18

**Authors:** Yingjie Zhou, Muhammad Tariq, Sijie He, Uzma Abdullah, Jianguo Zhang, Shahid Mahmood Baig

**Affiliations:** 1 Seven Section of Department of Gynaecology, The Second Hospital of Hebei Medical University, Shijiazhuang, Hebei China; 2 Human Molecular Genetics, Health Biotechnology Division, National Institute for Biotechnology and Genetic Engineering (NIBGE) College, PIEAS, Faisalabad, 38000 Pakistan; 3 BGI-Shenzhen, Shenzhen, 518083 China; 4 BGI Genomics, BGI-Shenzhen, Shenzhen, 518083 China

**Keywords:** Hearing loss, Whole exome sequencing, Consanguineous pedigrees, Clinical detection

## Abstract

**Background:**

Hearing loss is the most common sensory defect, and it affects over 6% of the population worldwide. Approximately 50–60% of hearing loss patients are attributed to genetic causes. Currently, more than 100 genes have been reported to cause non-syndromic hearing loss. It is possible and efficient to screen all potential disease-causing genes for hereditary hearing loss by whole exome sequencing (WES).

**Methods:**

We collected 5 consanguineous pedigrees from Pakistan with hearing loss and applied WES in selected patients for each pedigree, followed by bioinformatics analysis and Sanger validation to identify the causal genes.

**Results:**

Variants in 7 genes were identified and validated in these pedigrees. We identified single candidate variant for 3 pedigrees: *GIPC3* (c.937 T > C), *LOXHD1* (c.6136G > A) and *TMPRSS3* (c.941 T > C). The remaining 2 pedigrees each contained two candidate variants: *TECTA* (c.4045G > A) and *MYO15A* (c.3310G > T and c.9913G > C) for one pedigree and *DFNB59* (c.494G > A) and *TRIOBP* (c.1952C > T) for the other pedigree. The candidate variants were validated in all available samples by Sanger sequencing.

**Conclusion:**

The candidate variants in hearing-loss genes were validated to be co-segregated in the pedigrees, and they may indicate the aetiologies of hearing loss in such patients. We also suggest that WES may be a suitable strategy for hearing-loss gene screening in clinical detection.

## Background

Hearing loss is the most common sensory defect, and it affects ~ 1/500 newborns [1] and 466 million people worldwide (https://www.who.int/pbd/deafness/estimates/en/). Approximately 50% ~ 60% of hearing loss patients are attributed to genetic causes [1, 2]. Hereditary hearing loss is a genetically heterogeneous disorder [3] that can be divided into syndromic hearing loss and non-syndromic hearing loss, among which non-syndromic hearing loss is the predominant type, with a proportion of ~ 80% [4]. Currently, more than 100 genes have been reported to cause non-syndromic hearing loss (https://hereditaryhearingloss.org/), and the total number of genes related to hearing loss is expected to be several hundred.

There are mature gene panels for hearing-loss detection, and the genes involved range from 4 to more than 100. However, except for several genes, such as *GJB2* [5–7] or *SLC26A4* [8–10], most causal genes contribute a small fraction to the disorder. Therefore, in clinical detection, we may not obtain a satisfactory result by gene panel screening for many cases. As whole exome sequencing (WES) technology has rapidly developed and its cost has become less expensive, it is possible and efficient to screen all potential disease-causing genes for hereditary hearing loss by WES [11, 12].

Recessive inheritance hearing loss is worth studying because such patients usually have unaffected parents, which makes the disorder seem to be “sudden onset”, and this situation is more difficult to prevent. Consanguineous pedigrees represent a suitable natural model to study recessive disorders [13]. In Pakistan, there are numerous consanguineous pedigrees because of their customs, and these pedigrees may provide more opportunities to study and recognize such disorders [14, 15].

In this study, we collected 5 consanguineous pedigrees with hearing loss from Pakistan and applied WES to identify the causal genes. We identified several variants in hearing-loss genes that co-segregated in the pedigrees, and they may indicate the aetiologies of hearing loss in such patients.

## Methods

### Participants and clinical diagnosis

**Fig. 1:**
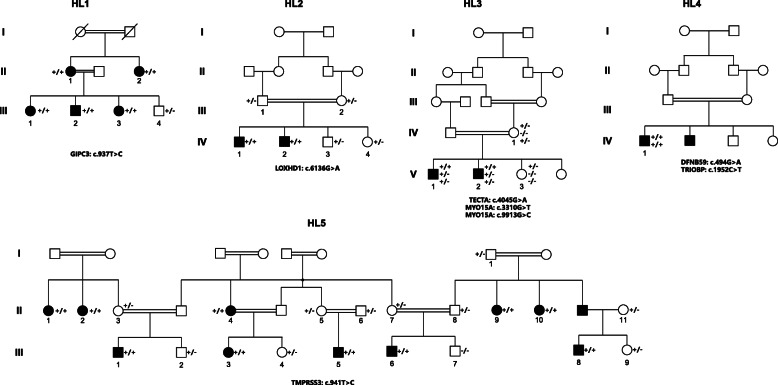
Pedigree figures of the five consanguineous families with genotypes of available individuals. The squares and the circles represent males and females, respectively. The black-filled symbols indicate patients with hearing loss, and a symbol with a diagonal line indicates a deceased family member. The symbols with numbers indicate the availability of the individual’s DNA. The candidate variants are listed under each pedigree, and the genotypes of the individuals for the variants are marked. “+/+” indicates homozygous variant, “+/−” indicates heterozygous variant, and “−/−” indicates reference

In the present study, we collected 5 consanguineous pedigrees containing 22 patients with hearing loss from rural areas in Pakistan. All the patients showed different degrees of hearing loss. The most likely inheritance mode for these pedigrees was autosomal recessive (Fig. 1). The study was approved by the ethical committee of the National Institute for Biotechnology and Genetic Engineering (NIBGE), Faisalabad, Pakistan, and all participants provided written informed consent (written informed consent of participants under the age of 16 were obtained from their parents or legal guardians).

### DNA extraction and whole exome sequencing

According to the manufacturer’s instructions, genomic DNA was isolated from the peripheral blood leukocytes of all participants using a DNA QIAamp mini kit (Qiagen, Hilden, Germany). One patient from each pedigree was selected, and WES was performed. Exons were captured using the BGI-Exome kit V4 and sequenced by BGI-seq 500 with 100 bp paired-end sequencing.

### Bioinformatics analysis

Low-quality reads were removed by SOAPnuke [16], and then the reads were mapped to the human genome reference (UCSCGRCh37/hg19) by the Burrows–Wheeler Aligner (BWA-MEM, version 0.7.10) [17]. Variants were called using the Genome Analysis Tool Kit (GATK, version 3.3) [18]. Variant Effect Predictor (VEP) [19] was used to annotate and classify all the variants. After that, all the variants were filtered based on their frequency in public databases (e.g., 1000 Genomes Project, Exome Sequencing Project and ExAC) and our in-house databases, and the variants with MAF < 0.005 were retained. Then, homozygous variants and compound heterozygous variants were selected because the most likely inheritance mode for these pedigrees was autosomal recessive. Finally, we applied several variant prediction tools including SIFT [20] (http://provean.jcvi.org/), PolyPhen2 [21] (http://genetics.bwh.harvard.edu/pph2/), MutationTaster [22] (http://www.mutationtaster.org/) and CADD [23] (https://cadd.gs.washington.edu/snv), to predict the functional impact of candidate variants [24].

### Sanger validation

DNA from all available samples in the five pedigrees was Sanger sequenced to validate the variants and confirm their co-segregation in the pedigree. Forward and reverse primers were designed by Primer3. After PCR amplification, the purified product was sequenced on ABI 3730XL DNA Analyzer.

## Results

### Clinical features

All the patients showed different degrees of hearing loss. In the first pedigree (HL1), all patients showed severe deafness, and one patient (III1) was selected for WES. In the second pedigree (HL2), all patients showed congenital profound deafness and muteness, and one patient (IV1) was selected for WES. In the third pedigree (HL3), all patients showed moderate deafness (their hearing loss started after seizures), and one patient (V1) was selected for WES. In the fourth pedigree (HL4), all patients showed congenital profound deafness, and one patient (IV1) was selected for WES. In the fifth pedigree (HL5), all patients showed moderate deafness, and one patient (III5) was selected for WES.

### Genetic analysis

WES was applied in the selected patients. The average depth of the target region was 146X with a coverage of 99.85%, and the coverage of the target region that was sequenced at least 10 times (depth > = 10 X) was 98.20% (Table 1). For each individual, more than ten thousand variants that may influence protein were identified. After frequency filtration (MAF < 0.005), approximately 15 ~ 32 exon variants were retained. Further inheritance model filtration retained 1 ~ 6 candidate variants (Table 1). All the rare variants detected in the exon region for the pedigrees are listed in the supplementary table 1. The original WES sequencing data of the samples were deposited in the CNSA (see “Availability of data and materials” section) and the samples information were listed in supplementary table 2.

**Table 1:** Sequencing and variants data

Pedigrees	HL1	HL2	HL3	HL4	HL5
Samples applied WES	III1	IV1	V1	IV1	III5
Sequencing depth (X)	136.98	136.47	142.36	143.74	170.79
Coverage (%)	99.75	99.88	99.9	99.83	99.9
10X coverage (%)	98.11	98.21	98.21	97.84	98.65
Exon variants with MAF < 0.005	23	22	32	15	19
Variants followed recessive model	1	4	6	3	1
Variants applied Sanger validation	1	1	3	0	1

We identified a stop codon lost homozygous variant, *GIPC3*: c.937 T > C, from the patient in the HL1 pedigree, and the variant prediction tools provided a benign prediction. For the patient from the HL2 pedigree, we identified 4 variants in 2 genes. However, one gene was reported to cause autosomal dominant hearing loss, therefore, we first analyzed the other gene. Then, a homozygous variant, *LOXHD1*: c.6136G > A, was regarded as a candidate variant for this pedigree. The variant prediction tools indicated a damaging prediction. For the patient from the HL3 pedigree, 6 variants in 5 genes were identified at first, and further analysis indicated that only 2 genes may cause autosomal recessive deafness. Therefore, the homozygous variant, *TECTA*: c.4045G > A, and two compound heterozygous variants, c.3310G > T and c.9913G > C in *MYO15A*, were regarded as candidate variants. The variant prediction tools indicated benign prediction for both *MYO15A* variants and damaging prediction for the *TECTA* variant. For the patient from the HL4 pedigree, 3 variants in 3 genes were identified, and two of them may cause autosomal recessive deafness. The two homozygous variants were *DFNB59*: c.494G > A and *TRIOBP*: c.1952C > T. The variant prediction tools indicated damaging prediction for the first variant and benign prediction for the last variant. For the patient from the HL5 pedigree, only one homozygous candidate variant was identified, *TMPRSS3*: c.941 T > C. The variant prediction tools indicated a damaging prediction. None of these variants were previously reported to cause hearing loss. The detailed information is listed in Table 2.

**Table 2:** Detailed information for candidate variants

Pedigree	Variant	RS_number	Gene	Strand	DNA change	AA change	Type	SIFT (score)	PolyPhen2 (score)	MutationTaster (score)	CADD (score)	ACMG classify
HL1	19–3,590,186-T-C	rs1466835034	GIPC3	+	c.937 T > C	p.*313Gluext*98	homozygous	NA	NA	Polymorphism	Benign	Likely pathogenic
								(NA)	(NA)	(not provided)	(11.43)	(PM2 + PM4 + PP1 + PP4)
HL2	18–44,063,569-C-T	rs774836161	LOXHD1	–	c.6136G > A	p.Glu2046Lys	homozygous	Damaging	Damaging	disease_causing	Damaging	Uncertain significance
								(0.016)	(0.995)	(not provided)	(29.2)	(PM2 + PP1 + PP3 + PP4)
HL3	11–121,016,765-G-A	rs141024429	TECTA	+	c.4045G > A	p.Ala1349Thr	homozygous	Tolerated	Benign	disease_causing	Benign	Uncertain significance
								(0.058)	(0.061)	(not provided)	(23.2)	(PM2 + PP1 + PP3 + PP4)
HL3	17–18,025,424-G-T	rs919809633	MYO15A	–	c.3310G > T	p.Gly1104Cys	compound heterozygous	Damaging	Benign	Polymorphism	Benign	Uncertain significance
								(0)	(0.011)	(not provided)	(15.33)	(PM2 + PP1 + PP3 + PP4)
HL3	17–18,069,800-G-C	rs535441567	MYO15A	–	c.9913G > C	p.Glu3305Gln	compound heterozygous	Damaging	Damaging	disease_causing	Damaging	Uncertain significance
								(0.007)	(0.83)	(not provided)	(27.9)	(PM2 + PP1 + PP3 + PP4)
HL4	2–179,320,814-G-A	NA	DFNB59	–	c.494G > A	p.Ser165Asn	homozygous	Damaging	Damaging	disease_causing	Damaging	Uncertain significance
								(0.02)	(0.931)	(not provided)	(25.2)	(PM2 + PP3 + PP4)
HL4	22–38,120,515-C-T	rs760246167	TRIOBP	+	c.1952C > T	p.Ser651Phe	homozygous	Damaging	Benign	Polymorphism	Benign	Uncertain significance
								(0.003)	(0.036)	(not provided)	(15.1)	(PM2 + PP3 + PP4)
HL5	21–43,795,850-A-G	NA	TMPRSS3	–	c.941 T > C	p.Leu314Pro	homozygous	Damaging	Damaging	disease_causing	Damaging	Uncertain significance
								(0.001)	(1)	(not provided)	(28.9)	(PM2 + PP1 + PP3 + PP4)

In summary, we identified one most likely causing variant for the HL1, HL2 and HL5 pedigrees and two most likely causing candidate variants for the HL3 and HL4 pedigrees.

### Sanger validation

**Fig. 2:**
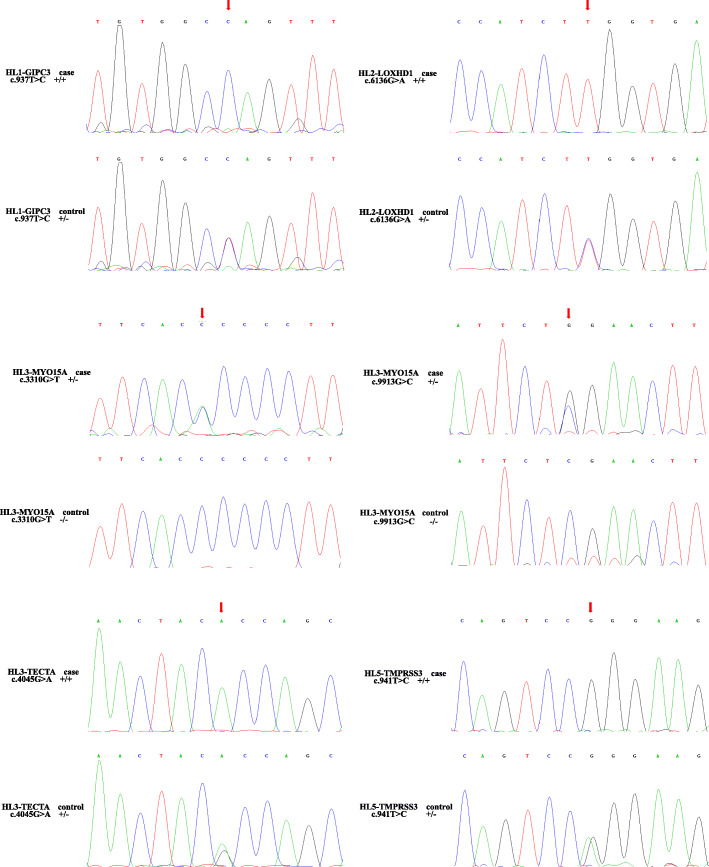
Sanger results of the candidate variants. The 6 variants are shown. Each variant contains a case and a control in the family. The Sanger result of the case is on the top, while the Sanger result of the control is on the bottom. The red arrow indicates the variant site

To validate co-segregation in the pedigree, we applied Sanger sequencing to all available samples. In total, 6 samples (II1 ~ 2 and III1 ~ 4) were sequenced for the HL1 pedigree, 6 samples (III1 ~ 2 and IV1 ~ 4) were sequenced for the HL2 pedigree, 4 samples (IV1 and V1 ~ 3) were sequenced for the HL3 pedigree, and 21 samples (I1, II1 ~ 11 and III1 ~ 9) were sequenced for the HL5 pedigree. For the HL4 pedigree, the initial samples collected were degraded, and we failed to collect additional samples. All the variants selected for Sanger sequencing were co-segregated in the pedigrees except for the HL4 pedigree (Fig. 2).

## Discussion

In this study, we identified several variants in genes reported to cause hearing loss that co-segregated in the pedigrees. For the HL1 pedigree, the variant in the *GIPC3* gene may cause the disorder. A protein with 312 amino acid residues is encoded by *GIPC3*, which contains a PDZ domain and three low-complexity regions [25]. The PDZ domain is responsible for the survival of hair cells and spiral ganglion in the ears. *GIPC3* was the causal gene of autosomal recessive deafness (type 15), non-syndromic genetic deafness and audiogenic seizures. Currently, 11 pathogenic variants in this gene have been identified in ClinVar.

For the HL2 pedigree, the variant in the *LOXHD1* gene was likely the causative variant. A highly conserved stereociliary protein is encoded by *LOXHD1*, which contains 15 PLAT domains that is responsible for protein interactions with the plasma membrane [26]. It was reported that *Loxhd1* maintained the cochlear hair cells’ function in mice [27]. *LOXHD1* was the causal gene of autosomal recessive deafness (type 77). Currently, 28 pathogenic variants in this gene have been identified in ClinVar.

For the HL3 pedigree, a homozygous variant in *TECTA* and two compound heterozygous variants in *MYO15A* were the likely candidate variants. A protein with 2155 amino acid residues is encoded by *TECTA*, which is a non-cellular matrix overlying the cochlear neuroepithelium. The function of this protein is to amplify and transmit sound [28, 29]. *TECTA* was the causal gene of autosomal recessive deafness (type 21), and 40 pathogenic variants in this gene have been identified in ClinVar. *MYO15A* encodes a protein that plays a crucial role in hair cells of the inner ear to maintain normal hearing [30]. *MYO15A* was the causal gene of autosomal recessive deafness (type 3), and 112 pathogenic variants in this gene have been identified in ClinVar. Take the inheritance mode of this pedigree (autosomal recessive) into consideration, we preferred that the homozygous variant in *TECTA* was more likely to be responsible than the compound heterozygous variants in *MYO15A*.

For the HL4 pedigree, homozygous variants were detected in both *DFNB59* and *TRIOBP*. A protein with 352 amino acids is encoded by *DFNB59*, and is responsible for the signal transmit of auditory nerve [31]. *DFNB59* was the causal gene of autosomal recessive deafness (type 59), and 9 pathogenic variants in this gene have been identified in ClinVar. A protein with 652 amino acids is encoded by *TRIOBP*, which regulates adherens junctions and recognizes actin cytoskeleton [32]. Currently, the function of *TRIOBP* remains unclear, and no pathologies except hearing loss were caused by pathogenic variants in this gene. *TRIOBP* was the causal gene of autosomal recessive deafness (type 28), and 26 pathogenic variants in this gene have been identified in ClinVar. It was predicted to be likely benign in the deafness variation database for the variant in *TRIOBP*. Therefore, we thought the variant in *DFNB59* was more likely to be the causal variant than the *TRIOBP* variant.

For the HL5 pedigree, the variant in *TMPRSS3* may cause the disorder. The protein encoded by this gene plays a crucial role in activating the ENaC sodium channel [33], and it regulates the Na^+^ concentration in the inner ear [34]. *TMPRSS3* was the causal gene of autosomal recessive deafness (type 8), and 23 pathogenic variants in this gene have been identified in ClinVar.

We calculated the density of reported pathogenic variants in these genes, which were 11.7/kb, 4.2/kb, 6.2/kb, 10.6/kb, 8.5/kb, 3.7/kb and 16.8/kb for *GIPC3*, *LOXHD1*, *TECTA*, *MYO15A*, *DFNB59*, *TRIOBP* and *TMPRSS3*, respectively. The density may indicate the degree of understanding or focus for different genes. Genes with low density, such as *LOXHD1* and *TRIOBP*, may have potential research value.

The majority of causal genes we identified for these pedigrees were not common hearing-loss genes. If we applied a common hearing-loss gene panel to screen these patients, we would obtain negative results, and the causal gene/variant for the patients would be missed. Therefore, WES may be a better strategy than panel sequencing for hearing-loss screening even in clinical detection.

## Conclusion

In conclusion, we applied WES in five consanguineous pedigrees (one patient per pedigree) from Pakistan with hearing loss, followed by Sanger sequencing for all available samples among the pedigrees to identify the causal genes for them. Several variants in hearing-loss genes were validated to be co-segregated in the pedigrees, and they may indicate the aetiologies of hearing loss in such patients. Moreover, we suggest that WES may be a suitable strategy for hearing-loss screening in clinical detection.

## Supplementary information

**Additional file 1.**

**Additional file 2.**

## Data Availability

The data that support the findings of this study have been deposited in the CNSA (https://db.cngb.org/cnsa/) of CNGBdb. The download link is http://ftp.cngb.org/pub/CNSA/data2/CNP0000508/, and the samples information can be found in supplementary table 2. The download links of public data used in this study are listed below. The human genome reference (hg19):ftp://hgdownload.soe.ucsc.edu/goldenPath/hg19/chromosomes/ 1000 Genomes Project:ftp://ftp.1000genomes.ebi.ac.uk/vol1/ftp/release/20130502/ Exome Sequencing Project: http://evs.gs.washington.edu/evs_bulk_data/ESP6500SI-V2-SSA137.GRCh38-liftover.snps_indels.vcf.tar.gz ExAC: http://gnomad.broadinstitute.org/downloads

## Abbreviations

ABI: Applied Biosystems
BGI: The Beijing Genomics Institute
BWA: Burrows–Wheeler Aligner
CADD: Combined Annotation Dependent Depletion
DFNB59: Autosomal Recessive Deafness Type 59
DNA: DeoxyriboNucleic Acid
ENaC: Epithelial sodium channel
ExAC: The Exome Aggregation Consortium
GATK: Genome Analysis Tool Kit
GIPC3: GIPC PDZ Domain Containing Family Member 3
GRCh37: The Genome Reference Consortium Human Genome Build 37
LOXHD1: Lipoxygenase Homology Domains 1
MAF: Minor allele frequency
MYO15A: Myosin XVA
NIBGE: National institute for Biotechnology and Genetic Engineering
PCR: Polymerase chain reaction
PDZ: PSD95/Dlg/ZO-1
PLAT: Plasminogen activator
SIFT: Sorting tolerant from intolerant
SOAP: Short Oligonucleotide Analysis Package
TECTA: Tectorin Alpha
TMPRSS3: Transmembrane Serine Protease 3
TRIOBP: TRIO And F-Actin Binding Protein
UCSC: University of California, Santa Cruz
VEP: Variant Effect Predictor
WES: Whole exome sequencing
